# SOCIAL VALUE OF THE ELDER PUMI MINORITY IN CHINA

**DOI:** 10.1093/geroni/igz038.2650

**Published:** 2019-11-08

**Authors:** Meng ran Gao

**Affiliations:** Peking University, Beijing, China

## Abstract

Social status of the elderly nowadays declines rapidly in China. As anthropologist Margaret Mead considered, post-figurative culture leading in contemporary society and the source of knowledge are from youth. The value of the elderly has been overlooked. However, in Pumi, one of the smallest ethnic minority groups in northwestern Yunnan Province of China, it is common that senior residents have high social status. This study examines the social values the Pumi elderly have by systematic analysis and participation observation methods. Based on data collected in a Pumi village during a 6-month fieldtrip, we conclude that Pumi elderly enjoy a high status in the community. They occupy core positions in all important ceremonies, such as religious activities and other daily activities including hospice. Factors behind the special old-age care phenomenon are Pumi’s history and its culture. The special culture has united the group members together and enhanced individual development with community social capital. It is clear that respecting elderly does not only contribute the transformation of ethnical knowledge but also enhance community cohesiveness. Evaluating the role of the elderly should not only from economic perspective, but also from the holistic perspective of social culture, so as to reconsider the importance of the elderly to our society.

